# Soil & Water Assessment Tool (SWAT) simulated hydrological impacts of land use change from temperate grassland to energy crops: A case study in western UK

**DOI:** 10.1111/gcbb.12628

**Published:** 2019-07-26

**Authors:** Amanda J. Holder, Rebecca Rowe, Niall P. McNamara, Iain S. Donnison, Jon P. McCalmont

**Affiliations:** ^1^ Institute of Biological, Environmental and Rural Sciences (IBERS) Aberystwyth University Aberystwyth UK; ^2^ Centre for Ecology & Hydrology Lancaster Environment Centre Lancaster UK; ^3^ College of Life and Environmental Sciences University of Exeter Exeter UK

**Keywords:** bioenergy, evapotranspiration, flooding, hydrology, *Miscanthus*, short rotation coppice, streamflow

## Abstract

When considering the large‐scale deployment of bioenergy crops, it is important to understand the implication for ecosystem hydrological processes and the influences of crop type and location. Based on the potential for future land use change (LUC), the 10,280 km^2^ West Wales Water Framework Directive River Basin District (UK) was selected as a typical grassland dominated district, and the Soil & Water Assessment Tool (SWAT) hydrology model with a geographic information systems interface was used to investigate implications for different bioenergy deployment scenarios. The study area was delineated into 855 sub‐basins and 7,108 hydrological response units based on rivers, soil type, land use, and slope. Changes in hydrological components for two bioenergy crops (*Miscanthus* and short rotation coppice, SRC) planted on 50% (2,192 km^2^) or 25% (1,096 km^2^) of existing improved pasture are quantified. Across the study area as a whole, only surface run‐off with SRC planted at the 50% level was significantly impacted, where it was reduced by up to 23% (during April). However, results varied spatially and a comparison of annual means for each sub‐basin and scenario revealed surface run‐off was significantly decreased and baseflow significantly increased (by a maximum of 40%) with both *Miscanthus* and SRC. Evapotranspiration was significantly increased with SRC (at both planting levels) and water yield was significantly reduced with SRC (at the 50% level) by up to 5%. Effects on streamflow were limited, varying between −5% and +5% change (compared to baseline) in the majority of sub‐basins. The results suggest that for mesic temperate grasslands, adverse effects from the drying of soil and alterations to streamflow may not arise, and with surface run‐off reduced and baseflow increased, there could, depending on crop location, be potential benefits for flood and erosion mitigation.

## Graphical abstract

Using the Soil & Water Assessment Tool (SWAT), we investigate changes to hydrology following land conversion of 50% and 25% of existing improved pasture in western UK to the bioenergy crop *Miscanthus* or short rotation coppice (SRC). Soil water content and streamflow were not significantly changed compared to the baseline scenario of existing land use. Surface run‐off was reduced for both crops (by up to 40%), whereas evapotranspiration was increased with SRC (by up to 5%), and reduced with *Miscanthus* (by up to 2%). Reductions in surface run‐off throughout the year could have potential benefits for flood mitigation schemes.

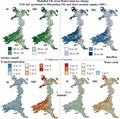

## 1 INTRODUCTION

Land use change (LUC) involving different crop types or management can influence ecosystem level hydrological processes. Quantification of these impacts is necessary to inform policy decisions based on trade‐offs between a range of potential positive and negative environmental impacts (DeFries & Eshleman, 2004; Foley et al., 2005; Mohr & Raman, 2013). The use of bioenergy crops for renewable energy generation can help to reduce reliance on fossil fuels and attain climate change objectives (Chum et al., 2011; CCC, 2018a). Although large‐scale uptake of dedicated energy crops in Europe has been slow to date (Lindegaard et al., 2016), their use as part of the energy generation mix is increasing (BEIS, 2018a) and renewable energy from biomass remains part of international and European climate mitigation policies (CCC, 2018b; IPCC, 2014). In Europe, as part of the long‐term strategy and vision for a ‘Climate neutral Europe by 2050’, sustainable expansion of bioenergy crops is likely to target economically marginal lands, avoiding any perceived competition with food crops whilst maximizing returns for land owners (CCC, 2018b; European Commission, 2018). However, the implication of this LUC for ecosystem hydrological processing is not fully understood, particularly for second‐generation (non‐food) bioenergy crops such as short rotation coppice (SRC; e.g. willow, *Salix* spp. and poplar, *Populus* spp.) and perennial grasses (e.g. switchgrass, *Panicum virgatum* L. and *Miscanthus*, *M. x* *giganteus*).

Temperate grasslands comprise a third of the utilized agricultural area across Europe and present a large potential area for the deployment of energy crops (Eurostat, 2018a). Changes in grazing management and reductions in agricultural subsidies, combined with typically poorer quality soils, are resulting in large areas of grassland becoming economically unprofitable (Donnison & Fraser, 2016; Eurostat, 2018b; Taube, Gierus, Hermann, Loges, & Schönbach, 2014). This is particularly noticeable for European regions such as Wales (UK) with a grass‐dominated agricultural landscape and a high proportion of land (80%) designated by the European Commission as ‘Less Favoured Areas’ (LFAs, agriculturally disadvantaged land in terms of soils, relief, aspect or climate, and receiving funding under the European Agricultural Fund for Rural Development, European Commission, n.d.).

Land suitability modelling suggests large areas (2,093 km^2^, 36% of west Wales) are suitable for bioenergy crops *Miscanthus* and SRC (Lovett, Sünnenberg, & Dockerty, 2014). Ambitious planting rates of up 50 km^2^/year have also been proposed as attainable with the potential for rural employment and diversification highlighted (ADAS UK Ltd [ADAS] & Energy Technologies Institute [ETI], 2016), which is especially relevant in the light of the uncertain future of UK (and indeed European) agricultural subsidies.

In comparison with grazed grassland, *Miscanthus* and SRC have the potential to impact on soil hydrological balance through an increased demand for water (Clifton‐Brown, Lewandowski, Bangerth, & Jones, 2002; Weih & Nordh, 2002), changes in root morphologies impacting water access through the soil profile (Crow & Houston, 2004; Neukirchen, Himken, Lammel, Czypionka‐Krause, & Olfs, 1999), differences in leaf development and morphology influencing evapotranspiration and precipitation interception (Finch & Riche, 2010; Holder, McCalmont, McNamara, Rowe, & Donnison, 2018; Stephens, Hess, & Knox, 2001), and taller, stronger stems changing hydraulic resistance to overland flows (Kort, Collins, & Ditsch, 1998; Marshall et al., 2009). As a result, there is generally an increase in evapotranspiration and a reduction in soil water recharge and surface run‐off, compared to existing land uses (Holder et al., 2018; McCalmont et al., 2017; Rowe, Street, & Taylor, 2009). These traits could be of benefit in landscape flood mitigation schemes (Environment Agency, 2015; Stephens et al., 2001) but can alter river flows and environments for aquatic and riparian species (Arthington, Naiman, McClain, & Nilsson, 2010; Poff & Zimmerman, 2010) and adversely affect dryland areas (Langeveld et al., 2012).

Resulting impacts of LUC to energy crops will be dependent on the extent of the area planted within river catchments and on regional climate, soil type, slope and altitude and stage of crop maturity (Hastings et al., 2014; Stephens et al., 2001; Vanloocke, Bernacchi, & Twine, 2010). This is reflected in previous studies of the impacts of land use conversions involving grassland to *Miscanthus* and SRC. For example, in modelled conversions from mixed land uses (grassland, corn and soybean) to *Miscanthus* in different regions of the American Midwest, Cibin, Trybula, Chaubey, and Brouder (2016) found that streamflow was reduced by around 8%, whereas Feng et al. (2018) found a mean reduction in streamflow of 23% (reflecting differing percentages of each land use type and varying topography). For SRC compared to conventional pasture, Hartwich et al. (2016) found that decreases in modelled surface run‐off varied from 20% to 78% in their study of the Northern German Plain with regional differences in climate and soils. These differences highlight the need for location‐specific modelling for the quantification of the potential impacts, positive or negative, of large‐scale bioenergy cultivation.

Hydrology simulation models linked to geographic information systems (GIS) can be used to gauge the effects of different LUC scenarios over varying spatial and temporal scales for specific locations, and a number of different models have been used in connection with biofuel scenarios (Engel et al., 2010; Finch et al., 2004; Vanloocke et al., 2010). The Soil & Water Assessment Tool (SWAT) is a physically based (i.e. representation of hydrological processes based on known principles of energy and water flux) hydrology model (Arnold, Srinivasan, Muttiah, & Williams, 1998) that can be incorporated into GIS software (Dile, Daggupati, George, Srinivasan, & Arnold, 2016). SWAT has been widely used to assess the impacts on hydrology and water quality of different land use management strategies (Engel et al., 2010) and has been successfully improved and used to represent *Miscanthus* and SRC crops (Hartwich et al., 2016; Trybula et al., 2015) enabling the use of the model for grassland LUC scenarios in Europe where the implications are unclear.

In this study, we aim to utilize the SWAT model with a GIS interface to quantify how water yield (amount of water leaving the catchment), soil water storage, evapotranspiration, surface run‐off, baseflow (groundwater flow) and streamflow respond to LUC from grassland to *Miscanthus* and SRC in a typical temperate agricultural grassland region at two planting levels: an ambitious ‘maximum’ (50% of available improved pasture) and more ‘limited’ (25% of improved pasture) level. Differences in responses between planting levels and bioenergy crop are also considered.

## 2 MATERIALS AND METHODS

### 2.1 West Wales River Basin and model description

**Figure 1:**
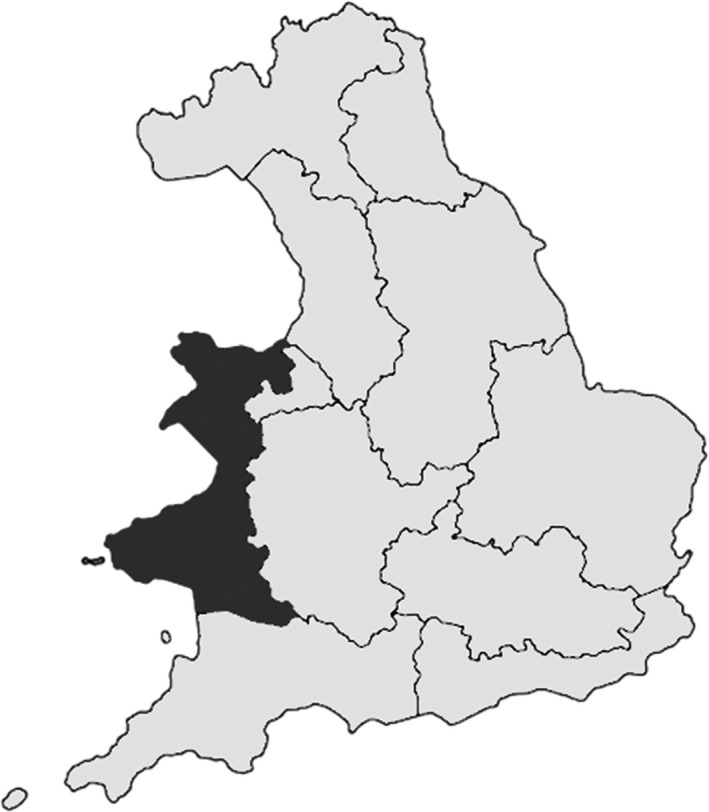
Environment Agency England and Wales Water Framework Directive river basin districts. The area covered by the West Wales River Basin used in this study is shown in black. This figure contains public sector information licensed under the Open Government Licence v3.0

The West Wales Water Framework Directive River Basin District (area 10,280 km^2^), hereafter referred to as the watershed (Figure 1; Environment Agency, 2014), is located in the western part of the UK and was chosen as a temperate region of Europe dominated by grass‐based agriculture and classed agriculturally as an ‘LFA’.

Hydrology for the watershed was modelled using the QSWAT v1.5 (rev. 664) extension with QGIS software (QGIS, 2014) and SWAT 2012 Editor interface (Arnold et al., 1998; Dile et al., 2016). A physical description of the watershed within the model (representing the baseline scenario of existing land use and conditions) was built up using the GIS layers detailed in Table 1.

**Table 1:** Description of data used within the SWAT hydrology model with source reference

Data type	Resolution	Source
Digital elevation model	50 m	OS Terrain 50 (Ordnance Survey, 2018)
Soil	1 km	Soil Parent Material (British Geological Survey Materials, 2018)
	5 km	The Digital Soil Map of the World v3.6 (UNFAO, 2003)
Land use	25 m	Land Cover Map 2015 (Rowland et al., 2017)
River network	15–30 m	OS Open Rivers (Ordnance Survey, 2018)
Inland water bodies	__	UK Lakes Portal ([Link])
	__	GB Lakes Inventory (NRW, 2018)
Streamflow	Seven locations	National River Flow Archive 2018 ([Link])
Climate	19 locations	National Centres for Environment Prediction ([Link])
	Four locations	Met Office climate data (Met Office, 2014)

Abbreviation: SWAT, Soil & Water Assessment Tool.

The United Nations Food and Agriculture Organisation's map (UNFAO, 2003), showing dominant soil types, was matched to the soil types given in the British Geological Survey soils map (British Geological Survey Materials, 2018) and the SWAT database soil codes. The watershed consists of mainly loamy soils with varying amounts of clay, silt and sand. Dystric Cambisols account for 50% of the area, Dystric Gleysols 23% and Gleyic Cambisols 19%. The remainder consists of small areas of Podzol (5%) and Humic Gleysols (2%). The watershed is predominately made up of low quality agricultural land (Welsh Government, n.d.), 40% of the watershed is >15% slope and 42% is >200 m a.s.l. (Ordnance Survey, 2018). The dominant agricultural land is improved grass pasture (52%), with only 4% of the area designated as arable or horticulture. Urban areas account for 3% of the watershed with the remainder of the land cover made up of natural grasslands (19%), woodlands (18%), and small pockets of heath and marsh (4%; Rowland et al., 2017).

The watershed was delineated into 855 sub‐basins based on the digital elevation model and river data. hydrological response units (HRUs) within each sub‐basin were divided based on soil type, land use and slope (divided into two bands, above and below 15%). Insignificant HRUs were excluded using the following threshold filters to ignore areas of less than: 10% land use; 20% soil class; and 10% slope band; and redistributed proportionally among those remaining (Dile, Srinivasan, & George, 2018).

**Figure 2:**
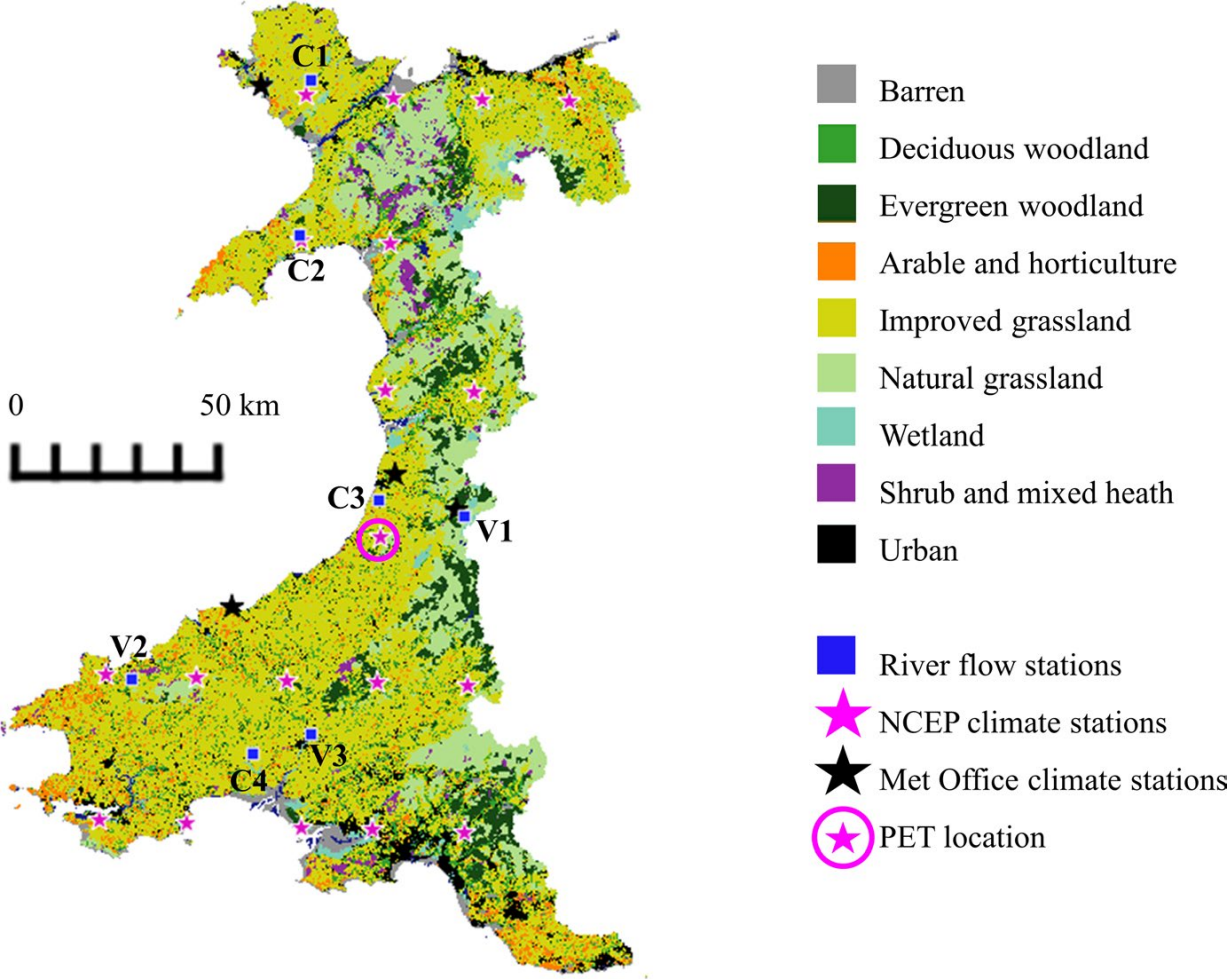
Land use as represented in the baseline Soil & Water Assessment Tool (SWAT) model for west Wales watershed (based on the Land Cover Map 2015, Table 1). Observed river flow from calibration (C1–C4) and validation (V1–V3) gauging stations was used to calibrate SWAT model predictions. Weather data were obtained from the National Centers for Environmental Prediction (NCEP) climate locations and UK Met Office climate stations. Potential evapotranspiration (PET) was calculated using data from the circled climate location

Climate data were obtained for 15 years from 1999 to 2013, the most recent period with all required data available (Table 1). The SWAT model was run on a monthly time step for the full duration using 1999 to 2003 as a 5 year warm up period (no results from the warm up period are used in the analysis). Climate data (precipitation, wind, relative humidity and solar radiation) obtained from the National Centers for Environmental Prediction (NCEP, n.d.) were checked for accuracy with long‐term weather data ranges using four UK Met Office climate stations (Met Office, 2014) located within the watershed (Figure 2). Mean annual precipitation in the watershed from 2004 to 2013 was 1,532 mm (Met Office, n.d.). Potential evapotranspiration (PET) was calculated with the r (R Core Team, 2015) package ‘Evapotranspiration’ Penman Monteith formula for short grass (Guo & Westra, 2016) using data from a representative weather station (Figure 2; Data S1) and read into the SWAT model (Neitsch, Arnold, Kiniry, & Williams, 2011). This resulted in the mean watershed PET being within estimates for the location and land cover type (based on Nisbet, 2005). Actual evapotranspiration was calculated within the SWAT model taking account of evaporation of canopy intercepted precipitation, crop transpiration and soil evaporation and sublimation, as detailed in the SWAT theoretical documentation (Neitsch et al., 2011).

The curve number (CN) method (USDA, 1986) was used in relation to simulation of surface run‐off within the model with adjustments allowed based on the steepness of the slope.

### 2.2 Plant growth simulation and management

In order to reflect expected growth rates for the region, plant inputs for the different land cover types were adjusted from the SWAT default values using values from the literature and, in the case of *Miscanthus*, some data was also obtained from measurements taken at a field‐scale trial site within the watershed. The main plant inputs used for the LUC crops and other land use cover plant types are shown in Tables 2 and 3, respectively. Arable agriculture in the watershed was based on typical crops grown in the region: wheat, barley, oats and oilseed rape (Welsh Government, 2018). Woodland biomass at the start of the simulations was input as 153 Mg DM/ha for evergreen forests and 136 Mg DM/ha for deciduous woodland (Forestry Commission, 2011, 2017).

**Table 2:** Main plant growth inputs for the land use change crops used in the simulations: Pasture (based on the SWAT land use code CRDY), *Miscanthus* and short rotation coppice. Values were taken from the SWAT database (SWAT: crop), measurements) or from the ranges suggested in the references. Where no reference is listed, a best estimation value was used

Input description	Pasture (CRDY)	*Miscanthus*	Short rotation coppice
Radiation use efficiency (kg ha^−1^/MJ m^−2^)	10 (Belanger, Gastal, & Warembourg, 1994; Cristiano, Posse, & Bella, 2015)	42 (Trybula et al., 2015) Measurements	28 (Bullard, Mustill, Carver, & Nixon, 2002; Linderson, Iritz, & Lindroth, 2007; Verlinden, Broeckx, Bulcke, Acker, & Ceulemans, 2013)
Max. stomatal conductance (m/s)	0.005 (SWAT: tall fescue)	0.005 (Beale, Bint, & Long, 1996; Clifton‐Brown & Lewandowski, 2000)	0.004 (SWAT: poplar)
Light extinction coefficient	0 (SWAT: tall fescue)	0.68 (Clifton‐Brown & Lewandowski, 2000)	0.5 (Linderson et al., 2007)
Max. leaf area index	4 (Asner, Scurlock, & Hicke, 2003)	11 (Trybula et al., 2015)	9 (Hartwich et al., 2016; Pellis, Laureysens, & Ceulemans, 2004; Schmidt‐Walter & Lamersdorf, 2012)
Min. leaf area index during dormancy	0.8	0 (Guo et al., 2018; Trybula et al., 2015)	0.75 (SWAT: poplar)
Max. canopy storage (mm)	0	2.2 (Stephens et al., 2001)	2.2 (Schmidt‐Walter & Lamersdorf, 2012; Stephens et al., 2001)
Max. canopy height (m)	0.75	3 Measurements	8 (Hartwich et al., 2016)
Max. root depth (m)	2 (SWAT: tall fescue)	2.5 (Neukirchen et al., 1999)	2 (Hartwich et al., 2016)
Optimum temperature (°C)	15 (SWAT: tall fescue)	20	15
Base temperature (°C)	0 (SWAT: tall fescue; Hurtado‐Uria, Hennessey, Shalloo, O'Connor, & Delaby, 2013)	8 (Hastings, Clifton‐Brown, Wattenbach, Mitchell, & Smith, 2009)	5 (Hartwich et al., 2016)

Abbreviation: SWAT, Soil & Water Assessment Tool.

**Table 3:** Main plant growth values used in the simulations for the land use types of arable (AGRL), lawn grass (BERM), natural grassland (FESC), evergreen forest (FRSE), heather/shrub grassland (MIGS), deciduous woodland (OAK), heather (SHRB) and fen/marsh/bog/saltmarsh (WETL). The model input variable name (Code) and references are shown where used (SWAT denotes the SWAT database)

Description	Code	AGRL	BERM	FESC	FRSE	MIGS	OAK	SHRB	WETL
Radiation use efficiency (kg ha^−1^/MJ m^−2^)	BIO_E	33.5 (SWAT)	10 (Belanger et al., 1994)	15 (Belanger et al., 1994; Cristiano et al., 2015)	15 (SWAT)	2 (Garbulsky et al., 2010)	2 (Garbulsky et al., 2010)	2 (Garbulsky et al., 2010)	5 (Garbulsky et al., 2010)
Max. leaf area index	BLAI	5 (Asner et al., 2003; AHDB 2018)	4 (SWAT)	4 (SWAT)	6 (Asner et al., 2003)	4 (Asner et al., 2003)	6.5 (Asner et al., 2003; ORNL DAAC, n.d.)	3.5 *(*Asner et al., 2003; Gonzalez et al., 2013)	5 (Asner et al., 2003)
Max. canopy storage (mm)	CANMX	0.8 (Wang, Li, & Rao, 2006)	—	1.2 (Burgy & Pomeroy, 1958)	3.7 (Hörmann et al., 1996)	1.5 (Dunkerley, 2000)	2.3 (Hörmann et al., 1996)	1.5 (Dunkerley, 2000)	1.2 (Burgy & Pomeroy, 1958)
Optimum temperature (°C)	TOPT	20 (Finch, Samuel, & Lane, 2002)	15 (SWAT: FESC)	15 (SWAT)	20	15 (SWAT: FESC)	15 (Bequet et al., 2011)	15	15
Base temperature (°C)	TBASE	5 (Finch et al., 2002)	0 (SWAT: FESC)	0 (SWAT)	0 (SWAT)	0 (SWAT: FESC)	5 (Bequet et al., 2011)	0	5
Fraction of tree biomass converted to residue	BIO_LEAF	—	—	—	0.0045 (Yang & Zhang, 2016)	—	0.003 (Yang & Zhang, 2016)	—	__
No. years to tree maturity	MAT_YRS	—	—	—	30 (SWAT)	—	100	—	__

Abbreviation: SWAT, Soil & Water Assessment Tool.

### 2.3 Miscanthus field measurements

A number of plant growth input values available in the literature for *Miscanthus* are based on measurements made in the American Midwest region from fertilized crops. Therefore, to check the suitability for their use in the region simulated in this project, the main *Miscanthus* growth values were checked using data obtained from an established *Miscanthus* plantation (~6 ha) located within the watershed. A full description of the field site (planted in 2012) and methods used for biomass sampling are given in McCalmont et al. (2017).

Mean annual harvest yields simulated by the model (14.74 Mg/ha, 2004–2013) were checked against the mean peak autumn yield (14.95 Mg/ha, 2014–2016, J. P. McCalmont, unpublished data) recorded at the site. The value used for radiation use efficiency (BIO_E: 41, Trybula et al., 2015) was found to be similar to an estimate of 42 made using measurements of photosynthetically active radiation and gains in *Miscanthus* above and belowground biomass between May 2015 and November 2016 (J. P. McCalmont, unpublished data).

Canopy height was recorded weekly during the 2017 growing season at eight randomly located measuring points within the crop (locations as shown in Holder et al., 2018) and reached a maximum of 3 m. Above ground biomass samples taken in February, June and August 2017 (from locations close to the eight measuring points) were freeze dried and subsequently ground to <2 mm using a Retsch mill (SM100; Retsch, Haan, Germany) before being further cryo‐milled in liquid nitrogen to a fine powder (6870 Cryomill; SPEX, Stan‐hope, UK). Samples were then analysed for total nitrogen (N) using a Vario Macro Cube Elementar (Analysensysteme GmbH, Langenselbold, Germany). Analysis of total phosphorus (P) was carried out by IBERS Analytical Chemistry (Aberystwyth, UK). This provided estimates of N and P at three seasonal time points (Table 4).

**Table 4:** Model inputs relating to *Miscanthus* above ground biomass nutrient contents (N, nitrogen; P, phosphorus) and residue decomposition rate. ‘Source reference’ details whether the value used for the SWAT model input (Code) was sourced from the literature (reference given) or derived from sampling at the field site within the watershed (measurement, with month samples taken)

Description	Code	Value	Source reference
Fraction N in yield	CNYLD	0.0032	Measurement (February)
Fraction P in yield	CPYLD	0.0005	Measurement (February)
Fraction N in biomass at emergence	BN1	0.024	Measurement (June)
Fraction N in biomass at 50% maturity	BN2	0.009	Measurement (August)
Fraction N in biomass at maturity	BN3	0.005	Guo et al. (2018); Ng, Eheart, Cai, and Miguez (2010); Trybula et al. (2015)
Fraction P in biomass at emergence	BP1	0.0024	Measurement (June)
Fraction P in biomass at 50% maturity	BP2	0.0016	Measurement (August)
Fraction P in biomass at maturity	BP3	0.0009	Trybula et al. (2015)
Plant residue decomposition coefficient (fraction)	RDSCO_PL	0.002	Amougou, Bertrand, Cadoux, and Recous (2012)

Abbreviation: SWAT, Soil & Water Assessment Tool.

### 2.4 Management operations

The following management operations were employed within the model depending on the land use/scenario for each HRU.

#### 2.4.1 Improved grassland

Sheep grazing at a stocking density of two livestock units starting in April for a duration of 212 days (to a minimum biomass of 1.5 Mg DM/ha; Genever & Buckingham, 2016). The daily dry weight of biomass eaten and trampled was set to 18 kg/ha (each), and fresh manure inputs to 60% of biomass consumed. Nitrogen fertilizer was added in March, April and July (40, 50, 20 kg N/ha respectively) and phosphorus was added in March, April and September (25, 15, 10 kg P/ha respectively; DEFRA, 2017). Pesticides were applied on a 2 year rotation: Year 1, Fluroxypyr MHE, Clopyralid and Triclopyr amine (0.32, 0.23, 0.42 kg/ha) were added in mid‐April based on the contents of Pastor^®^; Year 2, Glyphosate amine (0.54 kg/ha) was added at the beginning of October based on Roundup 360^®^ (Ballingall, 2014; Fera Science Ltd^2018^).

#### 2.4.2 Miscanthus

Fertilizer was automatically added by SWAT (according to crop N stress levels) to a maximum of 60 kg N ha^−1^ year^−1^ (amount required to obtain realistic yields within the model) and the above ground biomass was harvested annually in November at a 90% efficiency (based on field observations).

#### 2.4.3 Short rotation coppice

Fertilizer was automatically added by SWAT (according to crop N stress levels) to a maximum of 5 kg N ha^−1^ year^−1^ (being the amount required to obtain realistic yields within the model) and above ground biomass harvested in November on a 3 year rotation with a 70% efficiency (based on the SWAT database and Guo et al., 2015).

#### 2.4.4 Lawn grass

Fertilizer was automatically added to a maximum of 40 kg N ha^−1^ year^−1^. Grass was cut from April to August every 2 weeks, and then once a month during September and October.

#### 2.4.5 Arable

Fertilizer was automatically added to a maximum of 26 kg P ha^−1^ year^−1^ and 111 kg N ha^−1^ year^−1^ (DEFRA, 2017). All above ground biomass harvested (and plant growth killed) annually on 1 August (AHDB, 2018).

#### 2.4.6 Natural grassland

Light cattle grazing at a stocking density of 1.2 livestock units from mid‐May for a duration of 90 days (to a minimum biomass of 3 Mg DM/ha; Genever & Buckingham, 2016). The daily dry weight of biomass eaten and trampled was set as 22.5 kg/ha (each), and fresh manure inputs were 60% of biomass consumed. Beef fresh manure was also automatically added to a maximum of 25 kg ha^−1^ year^−1^ (DEFRA, 2017; Welsh Government, 2018).

### 2.5 Calibration

The initial model (representing existing land use) was calibrated for streamflow using the SWAT‐CUP 2012 v.5.1.6 Sequential Uncertainty Fitting (SUFI2) procedure (Abbaspour, 2015) and the protocol outlined in Abbaspour et al. (2015). Water flow calibration and validation stations were selected from the National River Flow Archive (NERC & CEH, n.d.), discarding those with outside factors that may influence flow (e.g. private ground water extraction). To achieve calibration, only watershed level parameters were amended (Table S2.1). Observed streamflow from gauging stations C1 to C4 (Figure 2) was compared to modelled streamflow from the relevant sub‐basin outlet and accuracy was assessed using *R* ^2^ and Nash–Sutcliffe efficiency results. Gauging stations located at V1–V3 (Figure 2) were used to validate the modelled streamflow data.

### 2.6 Scenarios

The baseline scenario is the calibrated model with existing land use. Four further simulations were run by splitting and changing the existing improved pasture land use and management to include the relevant percentage of energy crop (restricted to <15% slope, DEFRA, 2002; Lovett et al., 2014). *Miscanthus* planted on 50% (M50) and 25% (M25) and SRC planted on 50% (SRC50) and 25% (SRC25) of existing improved grass pasture within each sub‐basin. The maximum LUC scenario using 50% of existing pasture (2,192 km^2^) is based on the potentially suitable land in the district suggested in Lovett et al. (2014). The reduced, limited, level of LUC at 25% (1,096 km^2^) reflects a level that could be reached in ~20 years if potential ambitious planting schemes (ADAS & ETI, 2016) were taken up.

### 2.7 Analysis of results

Data analysis was performed in r version 3.5.1 (R Core Team, 2015) using linear models and linear mixed models (package ‘nlme’, Pinheiro, Bates, DebRoy, & Sarkar, 2017), with Tukey HSD (package ‘multcomp’, Hothorn, Bretz, & Westfall, 2008) post‐hoc tests for significant results. Model residual plots were checked for the appropriateness of each model. Linear mixed model results were summarized using type III ANOVA (package ‘car’, Fox & Weisberg, 2011) which performs a Wald chi‐square test.

For each level of planting, maximum (50%) or limited (25%), impacts of the crop type (baseline, *Miscanthus* and SRC) and season on the hydrological components of surface run‐off, baseflow, soil water content, evapotranspiration and water yield were explored using whole watershed means calculated for each month (2004–2013). For surface run‐off, baseflow and water yield transformations were used to improve model residuals (cube root with surface run‐off and square root with baseflow and water yield). Analysis was conducted separately for each planting level with models including crop type and month (and their interactions) as fixed factors and year as a random effect, with an auto correlation structure (AR1).

In addition, to compare between planting levels and bioenergy crop type, differences to the baseline (mm change in monthly means) were used. Linear mixed models included the fixed factors of LUC level (25% and 50%), crop type (*Miscanthus* and SRC), month, and the random effect of year and an auto correlation structure (AR1). Surface run‐off and baseflow data were transformed before testing (cube root and natural logarithm transformations respectively).

To allow for spatial effects to be examined, mean annual values (2004–2013) for all sub‐basins were produced and impacts on surface run‐off, baseflow, soil water content, evapotranspiration, water yield and streamflow were examined separately for each level of planting (50% or 25%) using linear models with crop type (SRC, *Miscanthus*, baseline) as a fixed factor. Streamflow data were transformed using the natural logarithm to improve residuals.

## 3 RESULTS

### 3.1 Model calibration

**Figure 3:**
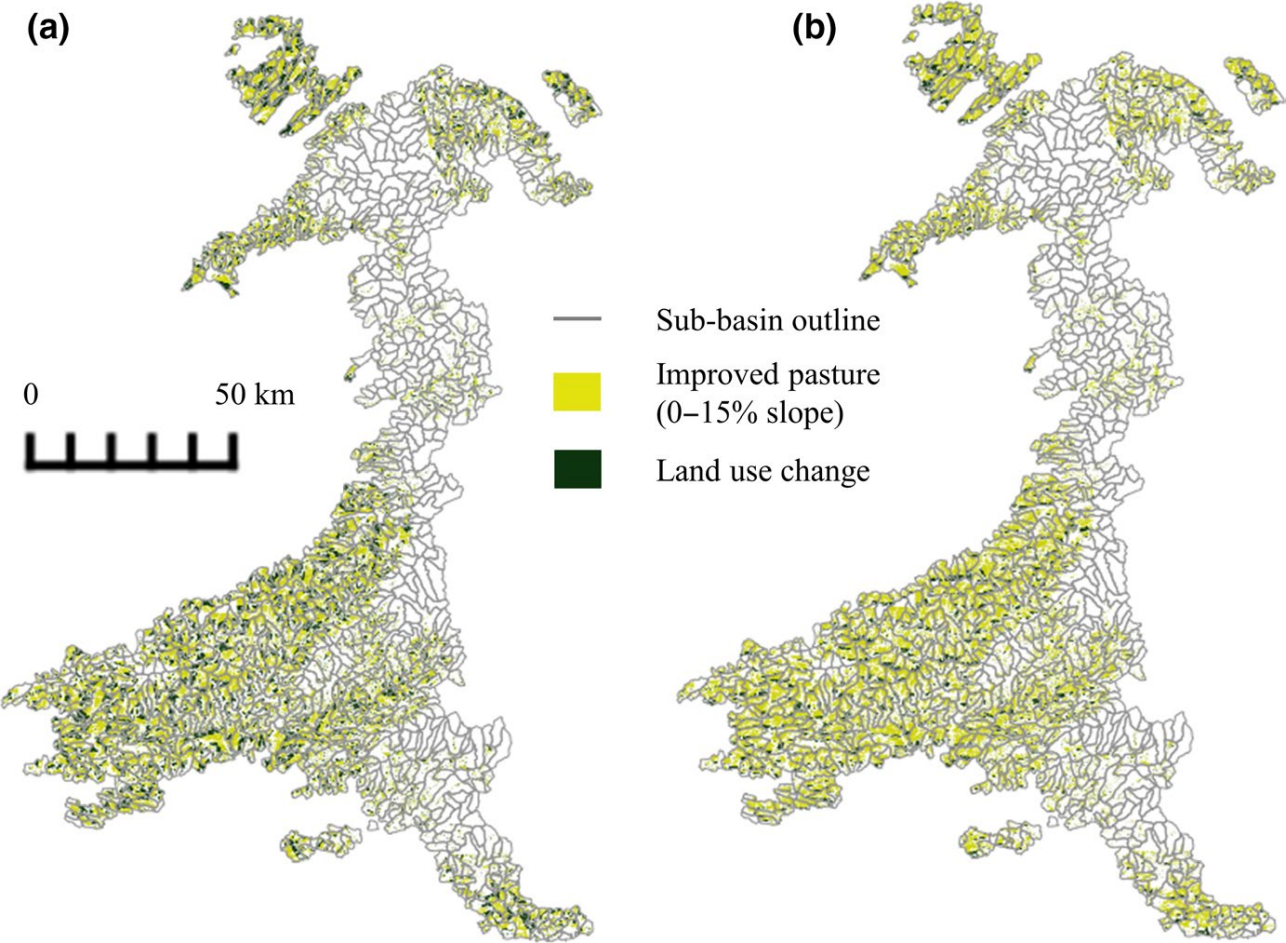
The West Wales River Basin District watershed delineated into 855 sub‐basins. The spread of the (a) maximum and (b) limited land use change scenarios (50% and 25%, respectively, of improved pasture in each sub‐basin) is represented

The watershed area was delineated into 855 sub‐basins (Figure 3) and 7,108 HRUs. Satisfactory calibration between observed and modelled streamflow was achieved with Nash–Sutcliffe efficiency coefficient values of >0.50 for the baseline scenario representing existing land cover (Table 5; Figure S2.1.1–S2.1.7). The CNs were increased from starting values for land in good hydrological condition in order to improve the correlation between observed and modelled streamflow. The final values used are shown in Table 6. Following amendments to plant growth parameters, simulated yields were checked against published data (Table 7; Figure S2.2.1–S2.2.4).

**Table 5:** Results of the correlation (*R* ^2^ and Nash–Sutcliffe [NS] values) between the observed streamflow at the calibration (C1–C4) and validation (V1–V3) locations (Figure 2) and the streamflow predictions for the relevant sub‐basin

Location	*R* ^2^	NS
C1	0.65	0.50
C2	0.73	0.67
C3	0.84	0.67
C4	0.83	0.81
V1	0.87	0.56
V2	0.76	0.59
V3	0.88	0.76

**Table 6:** Values used for the SWAT input codes (Code) controlling water erosion (USLE_C) and surface run off via Manning's N roughness coefficient (OV_N) and Soil Conservation Service Curve Number for each hydrological soil group (SCS A–D, USDA, 1986). Details shown are for the land use types of arable (AGRL), lawn grass (BERM), improved grass pasture (CRDY), natural grassland (FESC), evergreen forest (FRSE), heather/shrub grassland (MIGS), deciduous woodland (OAK), heather (SHRB) and fen/marsh/bog/saltmarsh (WETL). Source reference or SWAT database crop type are shown for the land use change crops of CRDY, *Miscanthus* (MSXG) and short rotation coppice (WSRC)

Code	AGRL	BERM	CRDY	FESC	FRSE	MIGS	MSXG	OAK	SHRB	WETL	WSRC
USLE_C	0.2	0.003	0.003 (SWAT: pasture)	0.003	0.001	0.003	0.003 (SWAT: Alamo)	0.001	0.003	0.003	0.001 (SWAT: poplar)
OV_N	0.14	0.1	0.15 (SWAT: pasture)	0.1	0.1	0.15	0.24 (Cibin et al., 2016)	0.14	0.15	0.05	0.14 (SWAT: poplar)
SCS_A	72	49	68	49	45	48	31	45	48	49	30
SCS_B	81	69	79	69	66	67	59	66	67	69	55
SCS_C	88	79	86	79	77	77	72	77	77	79	70
SCS_D	91	84	89 (Hess, Holman, Rose, Rosolova, & Parrott, 2010; USDA, 1986: grazed, no mulch)	84	83	83	79 (Cibin et al., 2016)	83	83	84	77 (USDA, 1986: trees, good)

Abbreviation: SWAT, Soil & Water Assessment Tool.

**Table 7:** SWAT simulated and reference mean biomass (for the month of August, 2004–2013) or yield (Y and harvest month) in dry mass units of Mg DM/ha. The SWAT database code used as the basis for each land use is shown; short rotation coppice (WSRC) and *Miscanthus* (MSXG) were added to the internal project database

Land use	Code	Simulated (*SD*)	Reference
Cereals/oil seed rape	AGRL	Y August: 4 (2.5)	7 Cereals, 3 oil seed rape (DEFRA, 2017)
Urban grass (mowed)	BERM	1.5 (0.4)	~4 cm sward height
Improved pasture (grazed)	CRDY	2.86 (2.6)	~2 depending on grazing strategy (Genever & Buckingham, 2016)
Natural grassland (light grazing)	FESC	3.5 (0.3)	3–7 (Mills, 2016); 1–3 (Milne, Pakeman, Kirkham, Jones, & Hossell, 2002)
Heather/shrub grassland	MIGS	9.75 (2.78)	6–27 (Mills, 2016); 5–10 (Milne et al., 2002)
Heather	SHRB	9.10 (2.26)	6–10 (Mills, 2016); 5–10 (Milne et al., 2002)
Fen/marsh/bog/saltmarsh	WETL	14.78 (10.74)	1–22 (Mills, 2016)
Short rotation coppice	WSRC	Y November: 13.71 (8.02)	5–16 (Aylott et al., 2008); 10–15 (Cunniff et al., 2015)
*M. x* *giganteus*	MSXG	Y November: 14.74 (9.92)	14 (Larsen et al., 2014); 15 measurements

Abbreviation: SWAT, Soil & Water Assessment Tool.

### 3.2 Effects at the West Wales River Basin watershed level

**Figure 4:**
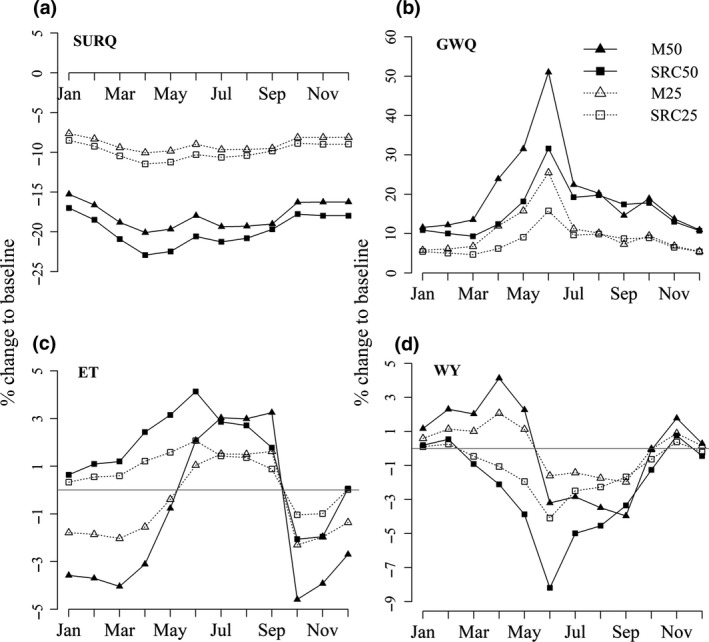
Percentage difference in the mean monthly (a) surface run‐off (SURQ), (b) baseflow (GWQ), (c) evapotranspiration (ET) and (d) water yield (WY), based on the 10 year simulation period, for each of the land use change scenarios compared to the baseline scenario of no land use conversion. The scenarios shown are *Miscanthus* (M50 and M25) and short rotation coppice (SRC50 and SRC25) planted on approximately 50% (2,192 km^2^) or 25% (1,096 km^2^) of improved pasture areas on or below a 15% slope

Impacts for the whole 10,280 km^2^ watershed varied across the months with the greatest differences occurring during the growing season (May–September, Figure 4). However, of the hydrological components tested (surface run‐off, baseflow, soil water content, evapotranspiration and water yield), only surface run‐off was significantly different compared to the baseline, where planting SRC at the 50% level resulted in significant reductions (*p* = 0.03) ranging from 17% (8 mm, January) to 23% (3 mm, April; Figure 4a).

Using the percentage change (compared to the baseline) to assess impacts of planting levels and bioenergy crop types, the 50% planting level (with both *Miscanthus* and SRC) led to greater reductions in overall surface run‐off than at the 25% level (*χ* ^2^(1) = 4.56, *p* = 0.03). In contrast, although the 50% planting level resulted in greater increases in baseflow than the 25% level (*χ* ^2^(1) = 49.94, *p* < 0.001), impacts were significantly different between the bioenergy crop types, where baseflow was increased more during the spring with *Miscanthus* than with SRC (*χ* ^2^(1) = 10.21, *p* = 0.001; Figure 4b).

The direction of change for evapotranspiration following LUC differed with bioenergy crop type, where it was increased with SRC during the early part of the year (January–May), but decreased with *Miscanthus* during the same period (*χ* ^2^(11) = 118.42, *p* < 0.001; Figure 4c). From October to December, both crop types showed a decrease following higher evapotranspiration over the growing season. Greater impacts generally resulted from the 50% planting level compared to the 25% level, although this also depended on crop species with greater differences found with *Miscanthus* than SRC (*χ* ^2^(1) = 10.86, *p* = 0.001).

Water yield showed a decrease during the growing season with both bioenergy crops; however, during the early part of the year, the *Miscanthus* crop resulted in an increase, which was in contrast to the decreasing trend with SRC (*χ* ^2^(11) = 27.85, *p* = 0.003). Impacts were again greater at the 50% planting level compared to the 25% but differences between crop types and planting levels were low from October to December (*χ* ^2^(1) = 10.92, *p* = 0.001).

### 3.3 Sub‐basin variation

**Figure 5:**
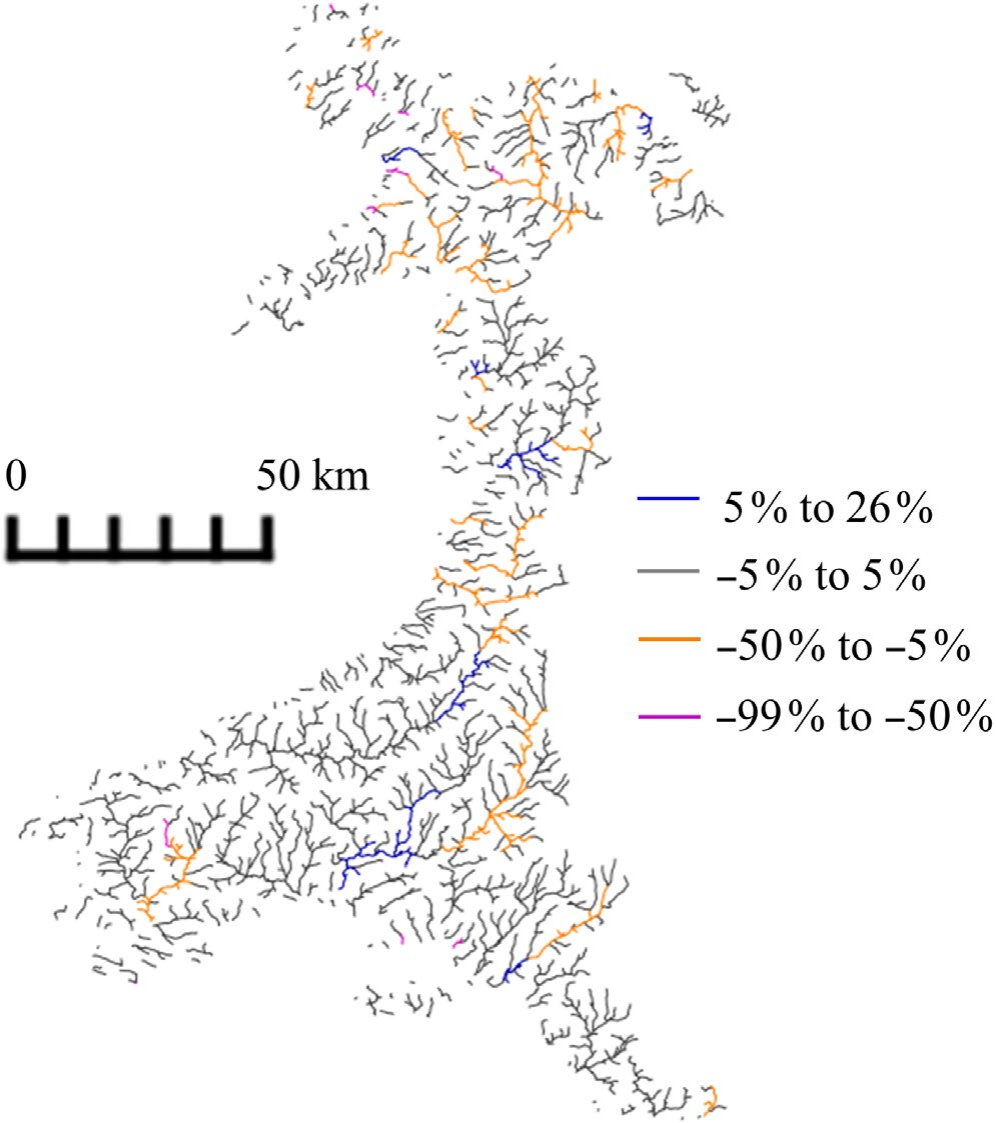
Mean percentage change in streamflow compared to the baseline. The change was the similar for each of the land use change (LUC) scenarios, and the percentage shown is the same for each crop type and LUC level

Land use change was simulated in 726 of the 855 sub‐basins (Figure 3), although it is also possible for non‐LUC sub‐basins to be impacted if, for example, they are downstream of the change. As changes in streamflow were limited in the majority of sub‐basins (Figure 5) and maximum changes in soil water content ranged from −3% to +2% across all the sub‐basins, these components were not found to significantly vary spatially (soil water content *F*_2,2562_ = 0.46, *p* = 0.63; *F*_2,2562_ = 1.83, *p* = 0.16; streamflow *F*_2,2562_ = 0.30, *p* = 0.74; *F*_2,2562_ = 0.38, *p* = 0.68; at the 25% and 50% levels respectively). However, reductions in streamflow of more than 50% were found in the same 10 sub‐basins for each LUC scenario. Streamflow in these 10 sub‐basins ranged from 0.5 to 1.6 m^3^/s (daily mean) in the baseline (existing land use) scenario.

**Figure 6:**
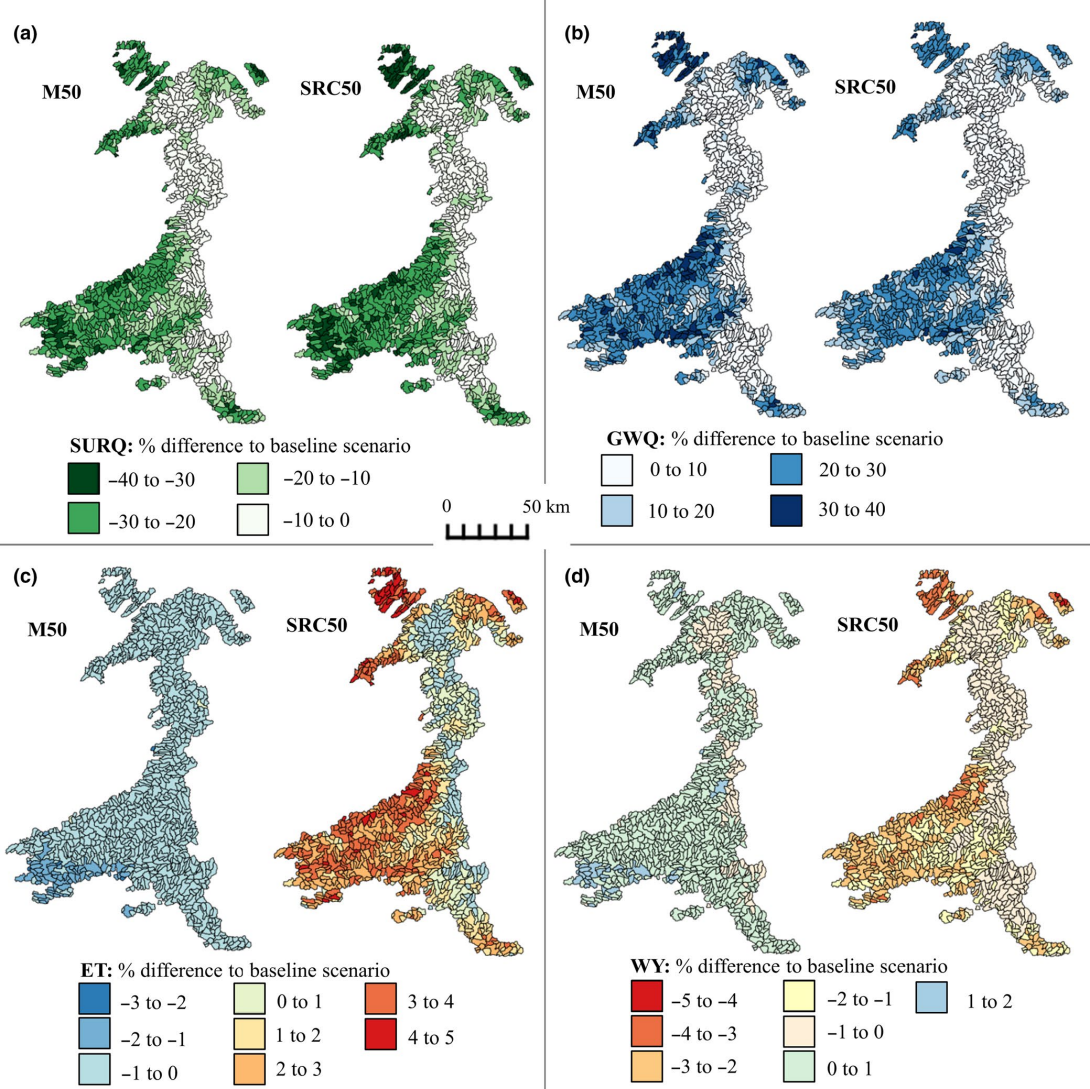
Percentage difference in mean annual (a) surface run‐off (SURQ), (b) baseflow (GWQ), (c) evapotranspiration (ET) and (d) water yield (WY) over the 10 year simulation period for the maximum land use change scenarios compared to the baseline case of no land use conversion. The scenarios shown are *Miscanthus* (M50) and short rotation coppice (SRC50) planted on approximately 50% (2,192 km^2^) of improved pasture areas on or below a 15% slope

The different LUC levels and crops had varying impacts on the other hydrological components (Figure 6; Table 8). Surface run‐off was significantly lower than the baseline scenario for *Miscanthus* and SRC in both the 25% (*F*_2,2562_ = 32.77, *p* < 0.001) and 50% (*F*_2,2562_ = 156.8, *p* < 0.001) scenarios, with differences ranging from 0 to −182 mm (0% to −40%, Figure 6a). No significant differences in surface run‐off were found between *Miscanthus* and SRC.

**Table 8:** Mean annual sub‐basin surface run‐off (SURQ), baseflow (GWQ), soil water content (SW), evapotranspiration (ET) and water yield (WY) in mm, and streamflow (daily mean, m^3^/s) for each of the scenarios (*SE* shown in brackets). The scenarios reflect planting *Miscanthus* (M) or short rotation coppice (SRC) on approximately 50% (2,192 km^2^) and 25% (1,096 km^2^) of existing improved pasture areas compared to the baseline (Base) of no land use change. Significance (*p* < 0.001) is shown for Base versus M/SRC

	Base (mm)	25%		50%	
		M	SRC	M	SRC
SURQ	344 (4)	314 (3)***	311 (3)***	284 (3)***	278 (3)***
GWQ	387 (2)	417 (2)***	413 (2)***	477 (2)***	439 (2)***
SW	166 (0.3)	166 (0.3)	166 (0.3)	167 (0.3)	167 (0.3)
ET	678 (1)	677 (1)	684 (1)***	676 (1)	691 (1)***
WY	851 (3)	852 (3)	845 (3)	853 (3)	838 (3)***
Flow out	1.27 (0.13)	1.25 (0.14)	1.24 (0.13)	1.25 (0.14)	1.24 (0.13)

Note

Significance denoted by ‘***’.

Baseflow results also showed greater differences in *Miscanthus* compared to SRC in the 50% LUC scenario where a significant difference (*p* = 0.02) was found between the two crops (Figure 6b). Eighty‐four sub‐basins in the M50 scenario increased baseflow by more than 30%, compared to 11 sub‐basins in the SRC50 scenario. The maximum amount of the increase was 39% (136 mm) for M50 and 36% (127 mm) for SRC50. Baseflow was significantly higher than the baseline scenario for both *Miscanthus* and SRC in the 25% (*F*_2,2562_ = 70.29, *p* < 0.001) and 50% (*F*_2,2562_ = 233.6, *p* < 0.001) LUC scenarios.

Changes in evapotranspiration with *Miscanthus* and SRC compared to the pasture baseline ranged from −2% (−15 mm, M50) to 5% (+32 mm, SRC50) and whilst the difference was only significant for SRC (*p* < 0.001), a distinct difference was seen between the two crops (*p* < 0.001). Where changes in evapotranspiration relating to the *Miscanthus* scenarios occurred, the result was a small reduction; however, with SRC increases were produced (Figure 6c). The same trend was identified in the 25% LUC scenarios. It was also found that some of the sub‐basins with the highest increase in evapotranspiration also had the highest reductions in water yield (Figure 6c,d).

Changes in water yield compared to the baseline scenario were not significant at the 25% LUC level. However, for the 50% LUC scenarios, SRC was significantly lower than both the *Miscanthus* (*p* = 0.001) and baseline (*p* = 0.01) scenarios (Figure 6d). Differences in water yield ranged from a reduction of 4% (−30 mm, SRC50) to an increase of 2% (+16 mm, M50).

## 4 DISCUSSION

This study has shown that large‐scale planting of *Miscanthus* or SRC crops does have a significant impact on the hydrological cycle for the West Wales River Basin. The simulated reductions in surface run‐off and increases in baseflow for *Miscanthus* and SRC (at the limited and maximum LUC levels) correspond with previous predictions relating to LUC to *Miscanthus* and SRC (Environment Agency, 2015; Stephens et al., 2001) where changes to these hydrological components followed a similar trend. The maximum monthly reduction (in mm) across the watershed for surface run‐off with *Miscanthus*, 17 mm (in November, a 17% reduction compared to the baseline scenario), was similar to the 18 mm maximum reduction simulated by Cibin et al. (2016) in modelled LUC from grassland to *Miscanthus* within a U.S. catchment. The 20%–30% reduction in surface run‐off found for the majority of the sub‐basins is also within the range of 20%–78% predicted by Hartwich et al. (2016) in modelled LUC from grassland to SRC (in different regions of the Northern German Plain).

It should be noted that the surface run‐off calculations used in the model simulations are based on the CN method (Soil Conservation Service, 1976) and Manning's roughness coefficients (e.g. Chow, 1959). These are well established for traditional crops, grassland and woodland but empirical measurements (to act as a basis for coefficient values) are lacking for *Miscanthus* and SRC (Environment Agency, 2015). The values we adopted for *Miscanthus* were previously used by Cibin et al. (2016) and are based on values for Alamo switchgrass (*P. virgatum* L.). Switchgrass is a similar perennial grass to *Miscanthus* but may exhibit morphological differences, for example an increased stem density compared to *Miscanthus* (Cassida, Muir, Hussey, & Read, 2005) that could result in differences in hydraulic resistance and hence surface run‐off rates. Similarly, new *Miscanthus* varieties (currently in pre‐commercial trials, Lewandowski et al., 2016) can have significantly different morphologies. SRC CNs used were based on existing values for trees, but an SRC plantation differs in stand layout and density compared to natural woodland and therefore (for both SRC and *Miscanthus*) empirical measurements would improve model inputs. However, whilst accuracy of the model could be improved in this respect, replacing grassland in comparison with grassland with the more rigid stems and greater height of both *Miscanthus* and SRC means that these crops would be expected to reduce run‐off and sediment flow.

Due to both physiological and physical factors (e.g. higher water use and greater leaf area index [LAI]), energy crops are generally associated with higher evapotranspiration than grassland, especially during the growing season (Cibin et al., 2016; Guo et al., 2018; Hartwich et al., 2016), something also found in this study. Differences in SRC compared to *Miscanthus* in evapotranspiration and water yield are slightly more complex. Whilst the longer SRC growing season can, in part, account for the greater impact of SRC than *Miscanthus*, modelled differences are also likely to be linked to specific parameters used for the LAI value during plant dormancy. In the *Miscanthus* scenarios this was set to zero (as in Trybula et al., 2015), whereas the LAI for the SRC scenarios during dormancy was set to 0.75 (as per the SWAT database for willow and poplar species). Although SRC and *Miscanthus* are not transpiring during winter months, LAI influences calculations of canopy storage and hence the evaporation of intercepted precipitation.

Whilst changes in water quality were not modelled, measured soil N losses following the establishment of *Miscanthus* and SRC have been found to reduce in comparison with annual crops and grassland due to lower fertilizer use and differences in N use efficiency (Christian & Riche, 1998; Schmidt‐Walter & Lamersdorf, 2012). Therefore, the reduction in fertilizer use with both *Miscanthus* and SRC (110, 60 and 5 kg N ha^−1^ year^−1^ for pasture, *Miscanthus* and SRC respectively) could be expected to reduce nitrate leaching. In addition, whilst the model required the addition of fertilizer to obtain expected crop growth based on published data (Aylott et al., 2008; Cunniff et al., 2015; Larsen, Jørgensen, Kjeldsen, & Lærke, 2014), fertilizer use is not routine in UK commercial production of these crops, particularly when cultivating on previously fertilized pasture land (Aylott et al., 2008; Terravesta Ltd, 2018). Fertilizer applications have been used in other SWAT‐based studies (e.g. 122 kg urea ha^−1^ year^−1^ with *Miscanthus*, Cibin et al., 2016, and 50 kg N ha^−1^ year^−1^ with willow, Wang, Jager, Baskaran, & Brandt, 2018) and although the best yield responses to N fertilization are generally achieved at around 60–100 kg N/ha, *Miscanthus* and SRC do not always show a response to fertilization (Aronsson, Rosenqvist, & Dimitriou, 2014; Cadoux, Riche, Yates, & Machet, 2012; Quaye & Volk, 2013).

The different rooting structures and water requirements of SRC and *Miscanthus* have the potential to cause drying of the soil profile under rain‐limited conditions (Donnelly, Styles, Fitzgerald, & Finnan, 2011; Stephens et al., 2001). Such drying could have negative impacts such as reductions in yields (Knapp, Briggs, & Koelliker, 2001; Richter, Riche, Dailey, Gezan, & Powlson, 2008) and changes in microbial processes and associated nutrient availability with implications for soil carbon stocks and greenhouse gas emissions (Jensen, Beier, Michelsen, & Emmett, 2003; Smith et al., 2008). However, such drying did not occur in either scenario modelled in this study with soil moisture levels remaining similar to the pasture baseline. This is in contrast to Hartwich et al. (2016) where soil water content was reduced in simulated LUC from pasture to SRC crops in the drier Northern German Plain, where soils are likely to have a higher sand content. Rainfall levels in west Wales (1,532 mm/year) are also towards the top end of the range (of between 1,000 and 1,600 mm/year) for areas including Ireland, western Great Britain, northern Italy, Switzerland, Austria and northern Spain (European Environment Agency, 2012). The soils in this study also have a high clay and silt content, factors that are likely to limit drying impacts compared to drier locations or free‐draining, lighter soils (Balogh et al., 2011; Marshall, Holmes, & Rose, 1996). Therefore, in assessing the land suitability for the cultivation of energy crops, local conditions should be considered to ensure rainfall rates are sufficient to meet crop demand (Richter et al., 2008). The fact that the majority of grasslands in Europe (as a fraction of total agricultural land area) tend to be located in wetter areas (Smit, Metzger, & Ewert, 2008) confirms that these locations should perhaps be targeted for this kind of agricultural diversification.

Reductions in the amount of water leaving the sub‐basins (water yield) were only significant for the maximum SRC LUC scenario, and changes in streamflow were not significant for any of the LUC scenarios. This indicates that changes in aquatic environments are likely to be limited across the whole watershed. However, some sub‐basins did show reductions in streamflow of over 50% which, when coupled with the difficulties in understanding and predicting biotic responses to altered flow rates (Bunn & Arthington, 2002; Shafroth et al., 2010), demonstrates the importance of local environmental flow assessments in proposed large‐scale energy crop planting (Poff et al., 2010). The significant reduction in surface run‐off and increase in baseflow found for both LUC levels and crop types could also impact on aquatic and riparian species (Gurnell, Bertoldi, & Corenblit, 2012), which should be considered when selecting suitable locations for energy crop deployment.

However, improvements in soil water infiltration seen in this study may also benefit flood mitigation by increasing soil water capacity during periods of high rainfall, as has been found with the use of young trees (<7 years old) in shelterbelts (Marshall et al., 2009). Although increases in baseflow were higher with *Miscanthus* than with SRC during the spring (possibly as a result of increased soil infiltration with *Miscanthus* due to the later leaf development), overall SRC in our modelling performed better than *Miscanthus* in terms of potential flood mitigation benefits. This is largely due to overall reductions in water yield (at the 50% LUC scenario) and increases in evapotranspiration (at both LUC levels). The annual *Miscanthus* harvest is also in contrast to SRC where the 3 year harvest cycle results in more overwinter standing plant material for 2 out of 3 years. However, the timing of the harvest for *Miscanthus* in the model was simulated as occurring in November, but *Miscanthus* can be (and often is in the UK) harvested as late as early spring where the presence of the senesced biomass continues to intercept precipitation (Holder et al., 2018), and tall stalks would provide further resistance to overland flows and may reduce some of the differences between the two crops.

Reductions in surface run‐off and increases in baseflow brought about by LUC can also act to slow and buffer high overland flows (Bronstert, Niehoff, & Brger, 2002; Marshall et al., 2009; OECD, 2016) with the predicted impact of slowing the flow rate across floodplains. This factor could therefore potentially release currently excluded land in flood zone areas for the planting of biomass crops (Environment Agency, 2015). In the scenarios we tested, slope was restricted to below 15% in order to allow for crop management and harvest, but if the crops were planted with the main aim of flood mitigation or nutrient buffering (e.g. as land margin buffer strips, Ferrarini et al., 2017) with less demand for commercial return, this assumption could be relaxed somewhat with the acknowledgment that annual harvest may sometimes be lost due to prevailing conditions preventing land access.

The large‐scale planting areas considered in this study were chosen to highlight the maximum effects of the land conversion scenarios. To set the more limited LUC scenario (1,096 km^2^) in context, it has the potential to provide 12%, 1,639 GWh (assuming a yield of 12 Mg DM/ha, Larsen et al., 2014; an energy content of 17.95 GJ/Mg DM, Felten, Fröba, Fries, & Emmerling, 2013; with a conversion efficiency of 25%, Nguyen & Hermansen, 2015) of the Welsh Government target for 70%, 13,431 GWh (BEIS, 2018b) of Welsh electricity consumed to come from renewables (National Assembly for Wales, 2017).

Specific locations for planting of energy crops within the watershed will ultimately be based on economic and social constraints and it is not likely that just *Miscanthus* or SRC would be grown but rather a mix chosen to suit local conditions and opportunities. Projections based on profitability (using existing farm scales and energy crop prices) have suggested a commercially viable planting area of 390 km^2^ of energy crops in Wales (Alexander et al., 2014). However, there is scope for this to increase (by as much as 300 km^2^/year across the UK) due to improvements in agronomy, changes to climate resulting in greater yields, boosts in demand, and increases in prices paid for supply or if incentivized with subsidies (ADAS & ETI, 2016; Alexander et al., 2014; Hastings et al., 2017). Overall, whilst there is potential for negative impacts in a small number of sub‐basins, this study shows that even with very ambitious levels of LUC the production of bioenergy crops within this catchment is unlikely to result in damaging impacts on basin‐level hydrological processes. The impacts on other ecosystem services however were not addressed and would need to be considered in any policies that seek to support large‐scale planting of energy crops.
