# Supplementary material for: Soil & Water Assessment Tool (SWAT) simulated hydrological impacts of land use change from temperate grassland to energy crops: A case study in western UK
Source: Glob Change Biol Bioenergy. 2019 Jul 26;11(11):1298–317. doi: 10.1111/gcbb.12628 (PMC6853257; doi:10.1111/gcbb.12628)
Supplement: Supplementary file 1 [file GCBB-11-1298-s001.docx]

**Supplementary Information**

**S1 Climate data**

Daily weather data (covering the period 1999-2013) from the National Centers for Environmental Prediction (NCEP) Climate Forecast System Reanalysis (CFSR) (NCEP, n.d.) were used in the SWAT model. Climate reanalysis (observations combined with a numerical model simulating coupled atmosphere-ocean-land systems) provides comprehensive global records of climate states for long time periods. CFSR data was checked against UK Met Office historic and long term averages at four stations located within the watershed (Fig. S1.1 – S1.5).

SWAT provides a choice of methods for calculating potential evapotranspiration (Priestley-Taylor, Hargreaves, and Penman-Monteith for a 40 cm tall reference crop of alfalfa) as well as the ability to read in externally calculated values (Neitsch *et al*., 2011). Calculating potential evapotranspiration (PET) within SWAT gave a mean watershed PET of 0.35 (fraction of precipitation) or below, whereas values of around 0.45 are more realistic for the region (Nisbet, 2005). PET was therefore calculated on a daily basis outside of SWAT (using R (R Core Team, 2015) package ‘Evapotranspiration’ Penman Monteith formula for short grass (Guo & Westra, 2016) and the values were read in. The daily climate data required was taken from a representative weather location (see main document Fig. 2). Use of the read in values resulted in an average PET for the watershed of 0.44. Actual evapotranspiration was calculated by SWAT.


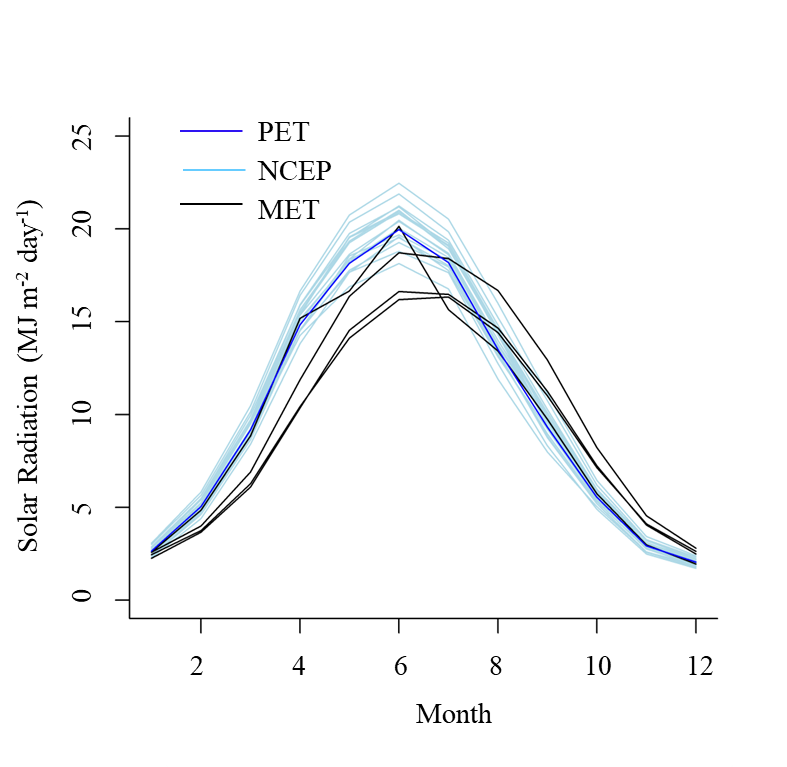


Fig S1.1 Mean daily solar radiation for each month from 1999 to 2013 for NCEP (National Centres for Environment Prediction) data and from 1999-2010 for the MET (UK Met Office) data. The PET line highlights the climate location used in potential evapotranspiration calculations.


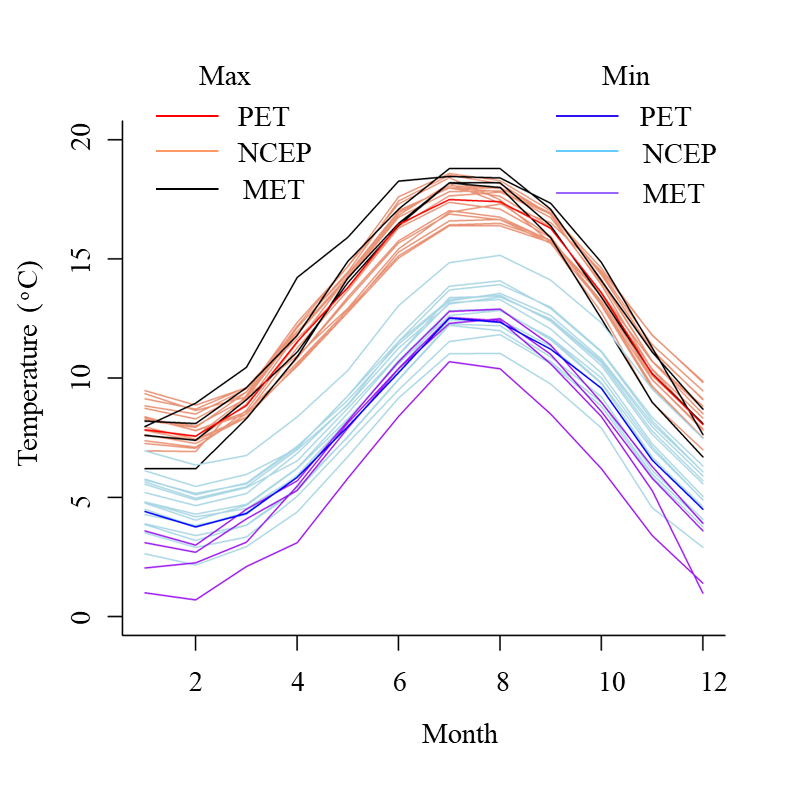


Fig S1.2 Mean daily maximum (Max) and minimum (Min) air temperature for each month from 1999 to 2013 for NCEP (National Centers for Environment Prediction) data and from 1999-2010 for the MET (UK Met Office) data. The PET line highlights the climate location used in potential evapotranspiration calculations.


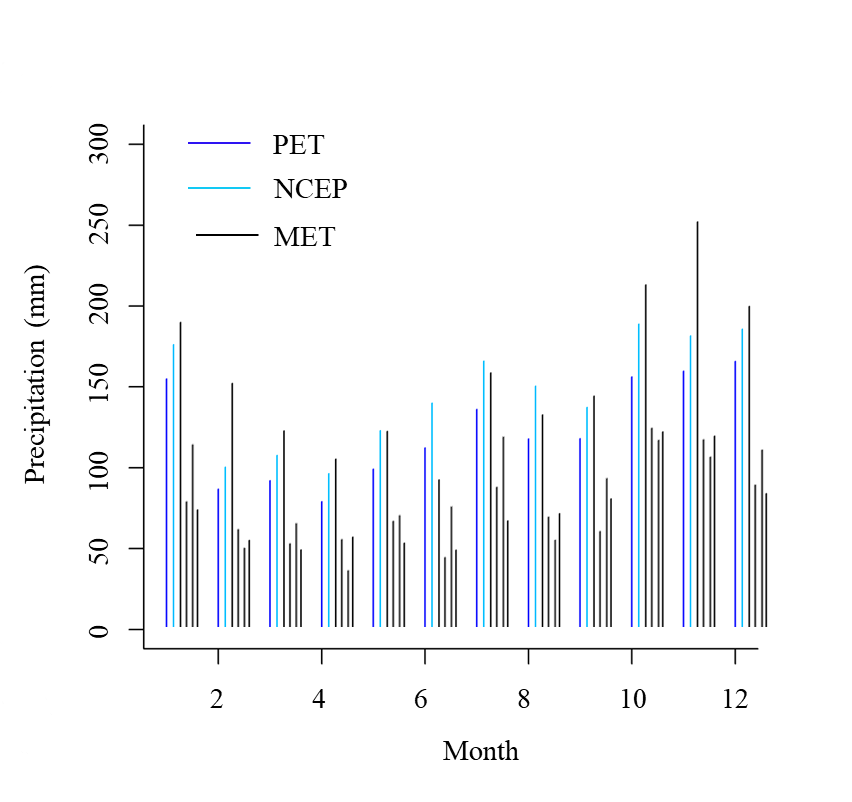


Fig S1.3 Mean total monthly precipitation from 1999 to 2013 for NCEP (National Centers for Environment Prediction) data (only highest value from NCEP locations shown) and from 1999-2010 for the MET (UK Met Office) data. The PET line shows the mean values for the climate location used in potential evapotranspiration calculations.


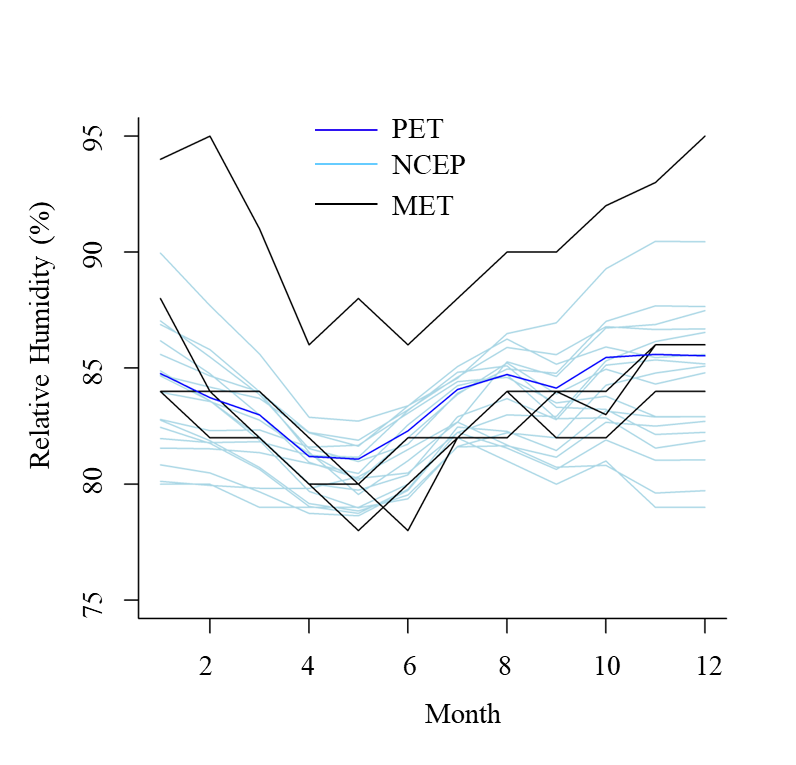


Fig S1.4 Mean daily relative humidity for each month from 1999 to 2013 for NCEP (National Centers for Environment Prediction) data and from 1999-2010 for the MET (UK Met Office) data. The PET line highlights the climate location used in potential evapotranspiration calculations. The station with the highest humidity is located at Gogerddan (52.43°N, 4.02°W).


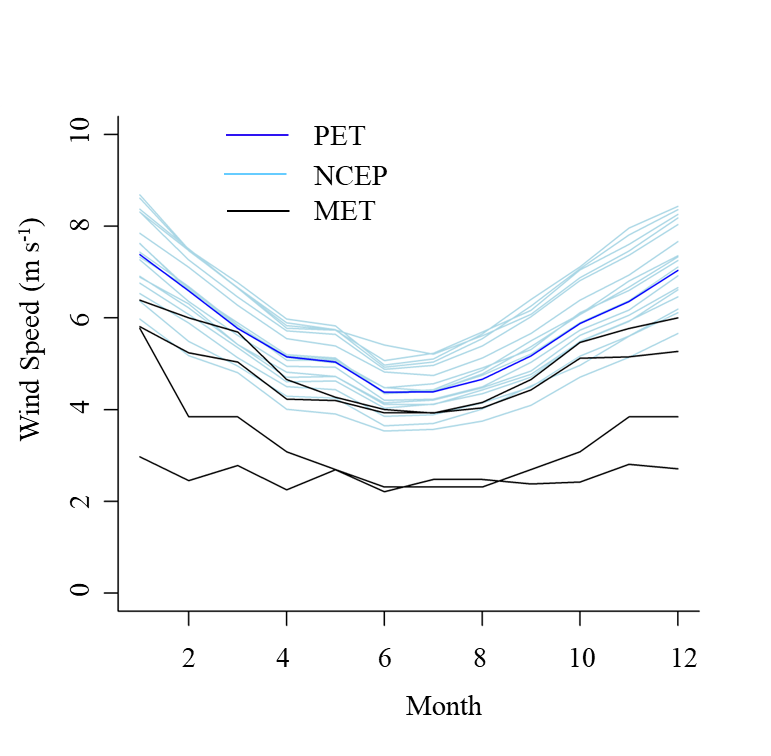


Fig S1.5 Mean daily wind speed in each month from 1999 to 2013 for NCEP (National Centers for Environment Prediction) data and from 1999-2010 for the MET (UK Met Office) data. The PET line highlights the climate location used in potential evapotranspiration calculations. The stations with the lowest wind speeds are located at Cwmystwyth (52.35°N, 3.80°W) and Gogerddan (52.43°N, 4.02°W).

**S2 Model calibration and validation**

**S2.1 Streamflow**

Calibration and validation of the baseline scenario (representing existing land use) was completed using SWAT-CUP 2012 v.5.1.6 Sequential Uncertainty Fitting (SUFI2) procedure (Abbaspour, 2015). 700 simulations were run with the adjustment of a selection of basin level parameters (Table S2.1) with the objective of achieving a Modified Nash Sutcliffe Efficiency (where *p*=3) of 0.5. The values used in the best simulation were used in all the modelled scenarios. Observed daily streamflow (NERC & CEH, n.d.) was converted to monthly mean values to correspond with the monthly time-step of the SWAT modelled streamflow outputs. Observed and modelled values were compared using R^2^ and Nash Sutcliffe coefficients to assess goodness of fit. Satisfactory calibration was achieved for all calibration and validation locations with a Nash Sutcliffe value of 0.50 or above (Fig. S2.1.1 – S2.1.7).

Table S2.1 SWAT input parameters (along with SWAT input code and file extension,(Arnold *et al.*, 2012)) adjusted with SWAT-CUP Sequential Uncertainty Fitting routines and the resulting best value ranges. The values used in the best simulation (objective of achieving a Modified Nash Sutcliffe Efficiency (*p*=3) of 0.5) were used in the model.

| **SWAT input parameter** | **Best range** | **Best simulation value** |
| --- | --- | --- |
| Threshold depth of water in shallow aquifer for “revap”/percolation  (REVAPMN .gw) | 961-994 | 978 |
| Groundwater “revap” coefficient  (GW_REVAP .gw) | 0.04-0.05 | 0.05 |
| Threshold depth of water to shallow aquifer for return flow  (GWQ_MN .gw) | 114-359 | 236 |
| Ground water delay time  (GW delay .gw) | 1.5-5.11 | 3.31 |
| Baseflow alpha factor  (Alpha_BF .gw) | 0.47-0.77 | 0.61 |
| Soil evaporation compensation factor  (ESCO .bsn) | 0.42-0.80 | 0.61 |
| Plant uptake compensation factor  (EPCO .bsn) | 0.57-0.87 | 0.72 |
| Surface runoff lag coefficient (SURLAG .bsn) | 0.16-23 | 12 |

Fig S2.1.1 Observed and modelled stream flow for location C1 (located on Anglesea at 53.26 °N and 4.35 °W). Correlation coefficients: R^2^ 0.65; Nash Sutcliffe Efficiency 0.50.

Fig S2.1.2 Observed and modelled stream flow for location C2 (located at Erch, 53.93 °N and 4.38 °W). Correlation coefficients: R^2^ 0.73; Nash Sutcliffe Efficiency 0.67.

Fig S2.1.3 Observed and modelled stream flow for location C3 (located at Ysywyth, 52.37 °N and 4.07 °W). Correlation coefficients: R^2^ 0.84; Nash Sutcliffe Efficiency 0.67.

Fig S2.1.4 Observed and modelled stream flow for location C4 (located at Dewi Fawr, 51.82 °N and 4.48 °W). Correlation coefficients: R^2^ 0.83; Nash Sutcliffe Efficiency 0.81.

Fig S2.1.5 Observed and modelled stream flow for location V1 (located at Cwmystwyth, 52.34 °N and 3.77 °W). Correlation coefficients: R^2^ 0.87; Nash Sutcliffe Efficiency 0.56.

Fig S2.1.6 Observed and modelled stream flow for location V2 (located at Gwaun, 51.97 °N and 4.90 °W). Correlation coefficients: R^2^ 0.76; Nash Sutcliffe Efficiency 0.59.

Fig S2.1.7 Observed and modelled stream flow for location V3 (located at Gwii, 51.87 °N and 4.28 °W). Correlation coefficients: R^2^ 0.88; Nash Sutcliffe Efficiency 0.76.

**S2.2 Crop growth time series**

Simulated growth for all land cover plants types were checked against literature values for expected growth within the region (see Table 7 in the main document). Examples of the crop growth patterns for the main three crops (taken from the same hydrological response unit (HRU)) used in each scenario are shown below (Fig. 2.2.1, 2.2.3 and 2.2.4). The early growth spurt and increased biomass for the grass pasture seen in 2012 is likely to be as a result of the warm winter with high air temperatures and solar radiation early in the year (Fig. 2.2.2) promoting a growth spurt before the start of grazing. Similar peaks are not seen with *Miscanthus* and SRC due to their later starting growing season.

Fig 2.2.1 Simulated improved pasture biomass growth (dry matter, DM). Sheep grazing is modelled from April to a minimum biomass of 1.5 Mg DM ha^-1^.

ͦͦͦ(°C)

(MJ M^-2^)

Fig 2.2.2 Average daily air temperature and solar radiation for the crop growth series shown in Figures 2.2.1, 2.2.3 and 2.2.4.

Fig 2.2.3 Simulated *Miscanthus* biomass growth (dry matter, DM) with an autumn harvest.

Fig 2.2.4 Simulated short rotation coppice (SRC) biomass growth (dry matter, DM) with autumn harvests on a three year cycle.

**References**

Abbaspour, K. C. (2015). *SWAT-CUP: SWAT Calibration and Uncertainty Programs - A User Manual*. Department of Systems Analysis, Integrated Assessment and Modelling (SIAM), Eawag. Swiss Federal Institute of Aquatic Science and Technology, Duebendorf, Switzerland. Retrieved from https://swat.tamu.edu/media/114860/usermanual_swatcup.pdf

Arnold, J. G., Kiniry, J. R., Srinivasan, R., Williams, J. R., Haney, E. B., & Neitsch, S. L. (2012). Input/Output Documentation. *Soil and Water Assessment Tool “SWAT,”* 1068–1068. Retrieved from http://link.springer.com/10.1007/978-0-387-35973-1_1231

Guo, D., & Westra, S. (2016). Evapotranspiration: Modelling Actual, Potential and Reference Crop Evapotranspiration. R package. Retrieved from https://cran.r-project.org/package=Evapotranspiration

National Centers for Environmental Prediction (NCEP). (n.d.). Global Weather Data for SWAT. Retrieved February 1, 2018, from http://www.waterbase.org/download_data.html

Natural Environment Research Council (NERC), & Centre for Ecology & Hydrology (CEH). (n.d.). National River Flow Archive 2018. Retrieved February 1, 2018, from https://nrfa.ceh.ac.uk

Neitsch, S. L., Arnold, J. G., Kiniry, J. R., & Williams, J. R. (2011). SWAT Theoretical Documentation Version 2009. *Texas Water Resources Institute*, 1–647. https://doi.org/10.1016/j.scitotenv.2015.11.063

Nisbet, T. (2005). Water Use by Trees - Forestry Commission Information Note FCIN065, 1–8. Retrieved from https://www.forestry.gov.uk/pdf/FCIN065.pdf/$file/FCIN065.pdf%0Ahttp://www.forestry.gov.uk/pdf/fcin065.pdf

R Core Team. (2015). R: A language and environment for statistical computing. R Foundation for Statistical Computing, Vienna, Austria. URL https://www.R-project.org/.
